# Trends in prescription opioid use and dose trajectories before opioid use disorder or overdose in US adults from 2006 to 2016: A cross-sectional study

**DOI:** 10.1371/journal.pmed.1002941

**Published:** 2019-11-05

**Authors:** Yu-Jung Jenny Wei, Cheng Chen, Roger Fillingim, Siegfried O. Schmidt, Almut G. Winterstein

**Affiliations:** 1 Department of Pharmaceutical Outcomes and Policy, College of Pharmacy, University of Florida, Gainesville, Florida, United States of America; 2 Center for Drug Evaluation and Safety, University of Florida, Gainesville, Florida, United States of America; 3 College of Dentistry, University of Florida, Gainesville, Florida, United States of America; 4 Pain Research and Intervention Center of Excellence, University of Florida, Gainesville, Florida, United States of America; 5 Department of Community Health and Family Medicine, College of Medicine, University of Florida, Gainesville, Florida, United States of America; 6 Department of Epidemiology, College of Medicine and College of Public Health & Health Professions, University of Florida, Gainesville, Florida, United States of America; UCSD, UNITED STATES

## Abstract

**Background:**

With governments’ increasing efforts to curb opioid prescription use and limit dose below the Centers for Disease Control and Prevention (CDC)-recommended threshold of 90 morphine milligram equivalents per day, little is known about prescription opioid patterns preceding opioid use disorder (OUD) or overdose. This study aimed to determine prescribed opioid fills and dose trajectories in the year before an incident OUD or overdose diagnosis using a 2005–2016 commercial healthcare database.

**Methods and findings:**

This cross-sectional study identified individuals aged 18 to 64 years with incident OUD or overdose in the United States. We measured the prevalence of opioid prescription fills and trajectories of opioid morphine equivalent dose (MED) prescribed during the 12-month period before the diagnosis. Of 227,038 adults with incident OUD or overdose, 33.1% were aged 18 to 30 years, 52.9% were males, and 85.0% were metropolitan residents. Half (50.5%) of the patients had a diagnosis of chronic pain, 32.7% had depression, and 20.3% had anxiety. Overall, 79,747 (35.1%) patients filled no opioid prescription in the 12 months before OUD or overdose diagnosis, with the proportion significantly increasing between 2006 and 2016 (adjusted prevalence ratio, 1.86; 95% CI 1.79–1.93; *P* < 0.001). Patients without (versus with) prescribed opioids tended to be younger males and metropolitan and Northeast US residents. Of 145,609 patients who filled opioid prescriptions, 5 distinct prescribed daily dose trajectories preceding diagnosis emerged: consistent low dose (<3 mg MED, 34.6%), consistent moderate dose (20 mg MED, 27.3%), consistent high dose (150 mg MED, 15.0%), escalating dose (from <3 to 20 mg MED, 13.7%), and de-escalating dose (from 20 to <3mg MED, 9.4%). Overall, over two-thirds of patients with OUD or overdose with prescription opioids were prescribed a mean daily dose below 90 mg MED before diagnosis. Major limitations include the limited generalizability of the study findings and lack of information on out-of-pocket drug spending, race/ethnicity, and socioeconomic status of participants, which prevents analyses addressing these characteristics.

**Conclusions:**

In this study, we found that absence of opioid prescription fills in the year before incident OUD or overdose diagnosis was prevalent, and the majority of the patients received prescription opioid doses below the risk threshold of 90 mg MED. An increasing proportion of high-risk patients could be missed by current programs solely based on opioid prescribing and dispensing information in this new era of limited access to prescription opioids.

## Abstract 2

Yu-Jung Wei and colleagues investigate prescription opioid use in the year prior to opioid misuse or overdose.

## Author summary

**Why was this study done?:**

- Although US federal and state governments are increasing efforts to curb opioid prescribing, little is known about prescription opioid patterns preceding opioid use disorder (OUD) or overdose.
- The aim of our study was to describe prescribed opioid fills and dose trajectories preceding an OUD or overdose diagnosis among US adults.

**What did the researchers do and find?:**

- We used 11-year data (2006–2016) from a large US commercial insurance database to assess the prevalence of opioid prescription fills and trajectories of opioid morphine equivalent dose (MED) prescribed during the 12-month period before an incident OUD or overdose.
- After analyzing data from more than 200,000 patients with incident OUD or overdose, we observed that 35.1% of the patients filled no opioid prescription in the 12 months before diagnosis, with the proportion significantly increasing from 28% in 2006 to 42% in 2016. Among patients with opioid prescription fills, 5 distinct prescribed daily dose trajectories preceding diagnosis emerged, with most adults (72%) having prescribed doses below the high-risk threshold of a 90 mg MED.

**What do these findings mean?:**

- The findings of this study suggest that an increasing proportion of patients with OUD or overdose could be missed by current programs solely based on opioid prescribed use and dose in this new era of limited access to prescription opioids.
- Further studies are needed that examine the relationship between absence of prescription opioid fills and overdoses from illicit opioids and the association of prescription opioid dose trajectories with incident OUD or overdose.

## Introduction

Opioid use disorder (OUD) has significant public health consequences including opioid-related overdose events and deaths [1]. An estimate of 2 million individuals in the US have OUD, with the majority being adults aged 18 to 64 years [2]. The prevalence of diagnosed OUD in adult populations has increased between 2006 and 2011 (from 0.07% to 0.19%) [3] and continued to rise each year thereafter [4].

Although the increased incidence of OUD and overdose has been largely linked to the increased availability of prescription opioids [5], recent evidence shows a decline in opioid prescribing since 2011 [6], which is inversely related to OUD and overdose trends [7]. Potential explanations include a possible transition from prescription opioids to heroin and fentanyl, which most recently have surpassed prescription opioids in their contribution to opioid deaths [4,8,9]. Indeed, a 2015 national survey showed 59.9% of adults with misuse of prescription opioids used them without a prescription, and 40.8% obtained their opioids from friends or relatives [10]. Importantly, the same survey also found that patients with opioid misuse or OUD identified uncontrolled pain as the predominant reason for opioid misuse, emphasizing that OUD remains one of the mainstream clinical problems [10].

Starting in 2010, some health plans and several state regulations limit opioid prescriptions by duration (e.g., maximum of 3- to 14-day supply for certain pain conditions) and by dose (i.e., maximum of 90 mg morphine equivalent dose [MED] per day) [11–13]. Both private and public insurance plans have launched programs that flag individuals at risk for OUD and overdose based on high-dose and chronic use of opioid prescriptions. To monitor inappropriate prescribing, almost all states (excluding Missouri) now have a prescription drug monitoring program to capture controlled substance prescriptions paid by third-party payers or in cash [14]. Yet, these policies and programs, which depend on prescription-dispensing data and do not capture opioids traded in street markets, have been criticized for their low sensitivity in identifying high-risk patients, especially in an era of increasing transition to illicit opioid use [15].

A concern has been raised as to whether current policies and programs related to prescription opioid restrictions may drive patients, especially the high-risk opioid users, to seek alternative prescription medications (e.g., stimulants) or illicit opioids to achieve pain control or to enhance euphoric effects [16]. Here, we conducted a descriptive study to understand changes in prescription opioid patterns among patients with OUD or overdose in the past decade, during which many policies and programs aimed at limiting opioid prescribing were implemented. Using a population-based health claims database from 2005 to 2016, we examined the trend in the proportion of patients across study years with a new diagnosis of OUD or overdose who had filled no opioid prescription during the year preceding the diagnosis. Among patients who filled opioid prescriptions prior to diagnosis, we examined prescribed opioid dose trajectories and described the associations of demographic characteristics with the various prescription opioid dose patterns. Empirical data on prescription opioid trajectories are important in understanding whether there are typical and atypical drug use patterns. This information may serve as a warning sign to alert clinicians in timely identification of high-risk opioid use that requires further management and intervention [17].

## Methods

### Study design and source

This cross-sectional study used 2005–2016 IBM Truven MarketScan Commercial Claims data that contain billing records for inpatient and outpatient encounters and pharmacy-filled prescriptions as well as demographic characteristics and enrollment status of more than 20 million beneficiaries enrolled in employer-sponsored health insurance plans annually. The University of Florida institutional review and privacy boards approved the study without requirements to obtain informed consent from the subjects because the data are deidentified. Data analyses were performed as per a prespecified protocol (S1 Text) between January and May 2018. This study followed the Strengthening the Reporting of Observational Studies in Epidemiology (STROBE) reporting guideline (S1 STROBE Checklist).

### Study sample

To assemble the cohort, we identified patients who were aged 18–64 years at the time of a new OUD or overdose diagnosis, defined as having no history of either diagnosis during the 12 months preceding the date of the first recorded OUD or overdose (i.e., index date). We identified patients with OUD as having at least 1 inpatient or outpatient encounter claim with an ICD-9-CM code of 304.0x, 304.7x, or 305.5x or an ICD-10-CM code of F11.xx and patients with opioid overdose as having an ICD-9-CM code 965.0x or E850.0 to E850.2 or an ICD-10-CM code of T40.0xx to T40.4xx (wherein the “xx” placeholders contained X1 or X4) or T40.601, T40.604, T40.691, or T40.694 in any diagnostic position [18]. When identifying patients with incident OUD or overdose, we excluded ICD-10-CM codes that indicated “in remission” (e.g., F11.x1) or “subsequent encounter” (e.g., F40, 0X1D) of OUD or overdose. Patients were also required to have continuous health plan enrollment for 12 months before the index date and have no cancer and hospice care during this 12-month period.

### Prescription opioid and its dose conversation

Prescription opioids captured through the MarketScan pharmacy files included agents approved for use in the US between 2005 and 2016 (S1 Table). We excluded injectable opioids primarily used in inpatient settings where dispensing information is unavailable because of capitation-based reimbursement, rectal dosage forms (which are rarely used), and buprenorphine because it is mostly used for OUD treatment. Tapentadol and opioid prescriptions with missing strength (both <1% of the opioid prescription claims) were included in the analysis of opioid prescription receipt before OUD or overdose but excluded in dose trajectory analysis because of the lack of a conversion factor for estimating MEDs [19].

We converted the dose of each prescribed opioid fill to MED using a standard formula—the quantity of opioids dispensed per day multiplied by the strength and the MED conversion factor [19]. We then calculated the mean daily MED in each month of the 12 months before diagnosis at the patient level by dividing the sum of the MED of all prescribed opioids dispensed during the monthly interval by 30 days.

### Study measures

We focused on 2 measures: no opioid prescription (that is, the proportion of patients with a new diagnosis of OUD or overdose who did not fill any opioid prescription in the 12 months preceding the diagnosis) and the 12-month trajectories of prescribed mean daily opioid doses, which were identified using a group-based trajectory model (GBTM).

### Demographics

We measured demographics that had previously reported associations with OUD or overdose [20–23], including age, sex, status of health benefits coverage (dependent versus employee), metropolitan residency (yes versus no), and US geographic region (categorized as Northeast, Northcentral, South, and West).

### Statistical analysis

We reported demographics among adults with OUD or overdose as a single cohort as well as 2 separate disease subcohorts. In each subcohort, demographics were described by patients with and without prescription opioid fills in the year before the diagnosis. Among patients filling opioid prescriptions, we further analyzed chronic use of prescription opioids, defined as 70 days or more in a 90-day period [24]; high-dose use, assessed in terms of a cautionary dose of 50 mg/day MED or greater and a high-risk dose of 90 mg/day MED or greater in any 30-day period; and type of opioid use, grouped as short-acting only, long-acting only, and both short- and long-acting dosage forms. None of the reported variables had missing values.

In the single cohort of adults with incident OUD or overdose, we plotted the proportion of patients with no opioid prescription fill in the year before the diagnosis by the year of the diagnosis and within specific age groups (i.e., 18–30, 31–40, 41–50, and 51–64 years). In October 2015, the number of opioid-related diagnosis codes changed from ICD-9-CM codes to ICD-10-CM, resulting in a dramatic increase in the number of individuals diagnosed with OUD or overdose after the ICD-10 coding system [25]. Thus, a sensitivity analysis was conducted using ICD-9-CM codes only to test secular trends between January 1, 2006, and September 30, 2015, in proportions of patients without opioid prescription fills in the year before diagnosis.

To examine associations between demographics (independent variables) and absence of prescription opioid fill (dependent variable) among patients with OUD or overdose, we conducted a multivariable modified Poisson regression model and expressed associations as prevalence relative ratios (PRRs) and their respective 95% confidence intervals (CIs) [26]. In the model, we also adjusted for diagnosis of depression, anxiety, and various pain conditions (see operationalization in S1 Text). To test secular trends, we included each calendar year as a dummy variable in the model. The coefficients of these yearly dummy variables represent changes in the proportion of OUD patients without opioid prescription fill for a given year compared with the reference year of 2006.

Among adults with incident OUD or overdose who filled opioid prescriptions in the preceding year, we used a GBTM to identify clusters of individuals based on their probability of following a similar longitudinal pattern for opioid dose filled each month for 12 months before the diagnosis [17]. Because the repeated monthly mean MED measure had a nonnormal distribution (clustering between 0 and 170 MED), to enable model convergence while retaining all MED data points, we applied natural log transformation to the MED measure and modeled log-MED as a censored normal distribution. In each disease cohort, we fitted the GBTMs with 1 to 7 classes and found that a model with 5 trajectories was optimal within the recommended criteria [17,27] (S2 Table). Demographics were described and compared across the 5 trajectory groups using chi-squared test.

Because GBTM groups patients with a similar pattern for opioid dosage and reports mean MED for that group, patient-level doses may vary within a group. To test whether group-based mean daily MED adequately characterized patient-level MED estimates, we calculated the proportion of patients with a mean daily dose of 90 mg/day MED or higher in any given month across the 5 trajectories. All analyses were performed using SAS 9.4 (SAS Institute), and all tests were two-sided with statistical significance set at *P* < .05.

## Results

Among 227,038 eligible adult patients with incident OUD or overdose between 2006 and 2016, 33.1% were aged 18 to 30 years, 52.9% were males, and 85.0% were metropolitan residents; 205,945 (90.7%) had a first OUD diagnosis, and 21,093 (9.3%) had a first overdose event (Table 1). In the year before OUD or overdose diagnosis, half (50.5%) of the eligible patients had a diagnosis of chronic pain, and 65.0% had musculoskeletal pain. One-third (32.7%) had depression, and one-fifth (20.3%) had anxiety. Among those with opioid prescription fills before the diagnosis, the majority (71.2%) received short-acting opioids only, and 26.7% received a combination of short-acting and long-acting formulations. One in 4 (30.0%) of patients with OUD or overdose had chronic use of prescription opioids. More than 1 in 3 (43.3%) received prescription opioids at daily doses higher than 50 mg MED, and 1 in 4 (28.8%) had 90 mg MED or higher at any month during the 12-month prediagnosis period. Within 3 months before the diagnosis, only 0.5% and 0.2% of the adult patients had a prescribed opioid dose greater than 50 mg and 90 mg MED, respectively.

**Table 1:** Characteristics and prescription opioid receipt of adults, overall and stratified by OUD and overdose and prescription opioid receipt within 12 months before OUD or overdose diagnosis.

	No. (%) of Adults				
Characteristic	Patients With Incident OUD or Opioid-Related Overdose	Patients With OUD		Patients With Overdose	
		With Opioid Prescriptions	Without Opioid Prescriptions	With Opioid Prescriptions	Without Opioid Prescriptions
Total sample size	227,038 (100)	133,131 (100)	72,814 (100)	14,160 (100)	6,933 (100)
**Age at diagnosis, y**					
18–30	75,191 (33.1)	28,912 (21.7)	39,372 (54.1)	3,170 (22.4)	3,737 (53.9)
31–40	40,093 (17.7)	26,353 (19.8)	10,577 (14.5)	2,258 (15.9)	905 (13.1)
41–50	48,428 (21.3)	33,583 (25.2)	10,310 (14.2)	3,492 (24.7)	1,043 (15.0)
51–64	63,326 (27.9)	44,283 (33.3)	12,555 (17.2)	5,240 (37.0)	1,248 (18.0)
**Male**	120,006 (52.9)	67,766 (48.6)	46,158 (63.4)	5,304 (37.5)	3,778 (54.5)
**Insurance holder status**					
Dependent	121,393 (53.5)	65,660 (49.3)	43,749 (60.1)	7,676 (54.2)	4,308 (62.1)
Employee	105,645 (46.5)	67,471 (50.7)	29,065 (39.9)	6,484 (45.8)	2,625 (37.9)
**Living in metropolitan area**	193,079(85.0)	111,758 (83.9)	63,544 (87.3)	11,776 (83.2)	6,001 (86.6)
**Region**					
Northeast	44,014 (19.4)	20,997 (15.8)	19,816 (27.2)	1,842 (13.0)	1,359 (19.6)
Northcentral	44,419 (19.6)	25,196 (18.9)	13,932 (19.1)	3,477 (24.6)	1,814 (26.2)
South	94,606 (41.7)	59,271 (44.5)	27,116 (37.2)	5,767 (40.7)	2,452 (35.4)
West	43,999 (19.4)	27,667 (20.8)	11,950 (16.4)	3,074 (21.7)	1,308 (18.9)
**Pain diagnosis**					
Chronic pain	114,639 (50.5)	92,121 (69.2)	13,244 (18.2)	8,301 (58.6)	973 (14.0)
Neuropathic pain	51,348 (22.6)	41,595 (31.2)	5,721 (7.9)	3,664 (25.9)	368 (5.3)
Musculoskeletal pain	147,557 (65.0)	109,080 (81.9)	25,255 (34.7)	10,974 (77.5)	2,248 (32.4)
**Mental disorders**					
Depression	74,160 (32.7)	46,329 (34.8)	20,165 (27.7)	5,791 (40.9)	1,875 (27.0)
Anxiety	45,993 (20.3)	29,107 (21.9)	12,714 (17.5)	3,087 (21.8)	1,085 (15.6)
**Prescription opioid treatment**^**a**^ **within 12 months before OUD or overdose**					
Type of opioid agents					
Short-acting only	104,846 (71.2)	94,837 (71.2)	-	10,009 (70.7)	-
Long-acting only	3,062 (2.1)	2,836 (2.1)	-	226 (1.6)	-
Both short- and long-acting	39,383 (26.7)	35,458 (26.6)	-	3,925 (27.7)	-
Chronic opioid use^a^^,^^b^	43,625 (29.6)	39,833 (29.9)	-	3,792 (26.8)	-
High opioid dose					
≥50 MME/day in any month	63,121 (43.3)^c^	57,677 (43.8)^d^	-	5,444 (38.8)^e^	-
≥90 MME/day in any month	41,971 (28.8)^c^	38,218(29.0)^d^	-	3,753 (26.8)^e^	-
**Prescription opioid treatment**^**a**^ **within 3 months before OUD or overdose**					
Type of opioid agents					
Short-acting only	79,196 (53.8)	71,680 (53.8)	-	7,516 (53.1)	-
Long-acting only	3,424 (2.3)	3,055 (2.3)	-	369 (2.6)	-
Both short- and long-acting	24,804 (16.8)	22,195 (16.7)	-	2,609 (18.4)	-
Chronic opioid use	57,030 (38.7)	52,400 (39.4)	-	4,630 (32.7)	-
High opioid dose					
≥50 MME/day	773 (0.5)^c^	715 (0.5)^d^	-	58 (0.4)^e^	-
≥90 MME/day	335 (0.2)^c^	312 (0.2)^d^	-	23 (0.2)^e^	-

^a^The denominator was 147,291 patients who received at least 1 prescription opioid.

^b^Chronic opioid use was defined as using prescription opioids for 70 days or longer in a 90-day period.

^c^The denominator was 145,609 patients who had valid prescription opioid dosage converted to MME.

^d^The denominator was 131,582 patients who had valid prescription opioid dosage converted to MME.

^e^The denominator was 14,027 patients who had valid prescription opioid dosage converted to MME.

Abbreviations: MME, morphine milligram equivalent; OUD, opioid use disorder

**Fig 1:**
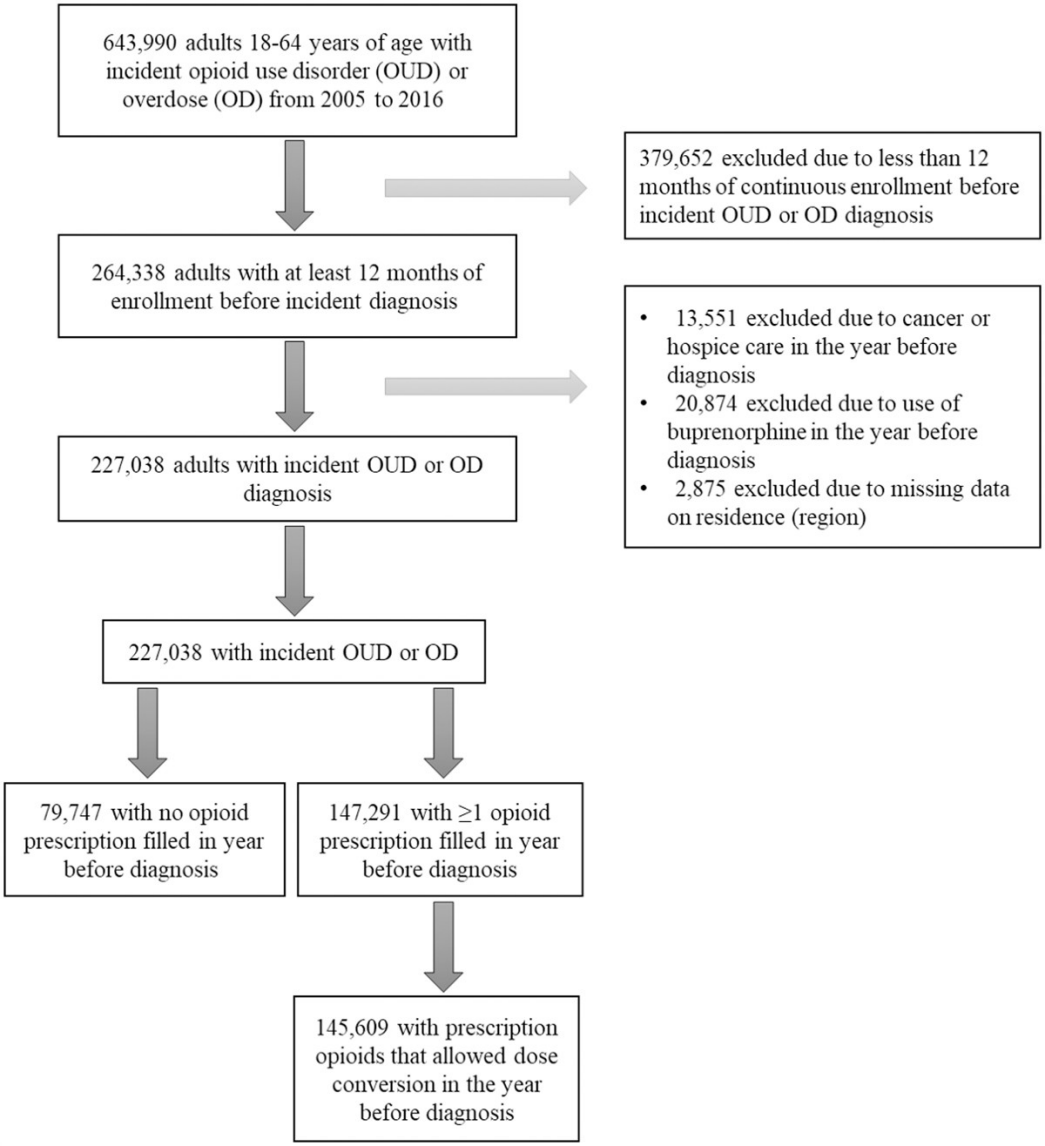
Flowchart of included patients.

**Fig 2:**
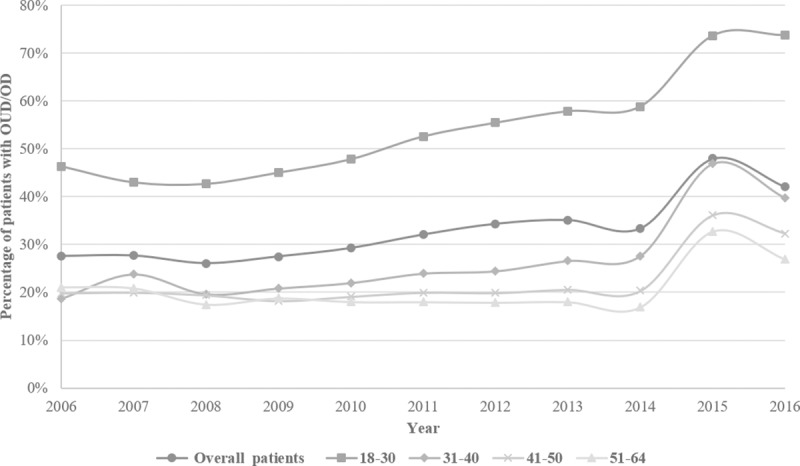
Secular trends in the proportion of study adults without prescription opioid fills within 12 months before OUD or OD diagnosis, overall and by specific age group. In each subfigure, lines represent trends, overall and within age groups, and for each line, each point represents annual percentage of patients with OUD or OD who had no opioid prescriptions. OD, overdose; OUD, opioid use disorder.

In the cohort of patients with incident OUD or overdose, over one-third (35.1%, 79,747/227,038) filled no opioid prescriptions within 12 months before the diagnosis (Fig 1). The crude (unadjusted) proportion increased from 28% in 2006 to a peak of 48% in 2015 (Fig 2). The overall increase was driven primarily by a 2-fold increase in the proportion among adults aged 31 to 40 years (19% in 2006 to 40% in 2016) and a 1.5-fold increase among younger adults aged 18 to 30 years (46% in 2006 to 74% in 2016). Data from the sensitivity analysis restricting the study period to the ICD-9-codes era only showed a similar consistent upward trend with a steeper slope in the younger age groups (S1 Fig).

Table 2 gives the associations between demographics and no opioid prescription fill in the year before diagnosis of OUD or overdose. After adjustment, we observed a significantly increasing proportion of patients with OUD or overdose who had no opioid prescription fills, starting in 2010 (PRR, 1.08; 95% CI 1.03–1.12; *P* < 0.001 versus 2006) and continuing through 2015 (PRR, 1.97; 95% CI 1.90–2.05; *P* < 0.001 versus 2006), followed by a slight decrease from the 2015 level in 2016 (PRR, 1.86; 95% CI 1.79–1.93; *P* < 0.001 versus 2006). Patients who were male, younger, metropolitan residents, and living in the Northcentral or Northeast areas were more likely to have no opioid prescription in the year before diagnosis.

**Table 2:** Associations between patient characteristics and no opioid prescription fills within 12 months before OUD or overdose diagnosis (*n* = 227,038).

Variables	Patients Without (Versus With) Any Prescribed Opioid Fills	
	Unadjusted PRR (95% CI)	Adjusted^a^ PRR (95% CI)
**Year (versus 2006)**		
2007	1.00 (0.95–1.06)	1.01 (0.96–1.05)
2008	0.95 (0.90–1.00)	0.98 (0.94–1.03)
2009	1.00 (0.95–1.05)	1.04 (1.00–1.08)
2010	1.06 (1.01–1.11)	1.08 (1.03–1.12)
2011	1.16 (1.11–1.22)	1.14 (1.09–1.18)
2012	1.25 (1.19–1.30)	1.20 (1.16–1.25)
2013	1.27 (1.22–1.33)	1.24 (1.19–1.29)
2014	1.21 (1.16–1.27)	1.27 (1.23–1.32)
2015	1.74 (1.67–1.82)	1.97 (1.90–2.05)
2016	1.53 (1.46–1.59)	1.86 (1.79–1.93)
**Age, y**	0.90 (0.90–0.91)	0.95 (0.94–0.95)
**Age**^**2**^**, y**	1.00 (1.00–1.00)	1.00 (1.00–1.00)
**Male (versus female)**	1.49 (1.48–1.51)	1.14 (1.13–1.15)
**Dependent (versus employee insurance status)**	1.32 (1.30–1.34)	0.92 (0.91–0.94)
**Metropolitan residency (yes versus no)**	1.20 (1.18–1.22)	1.06 (1.04–1.07)
**Region (versus South)**		
Northcentral	1.13 (1.12–1.15)	1.04 (1.03–1.06)
Northeast	1.54 (1.52–1.56)	1.22 (1.21–1.24)
West	0.96 (0.95–0.98)	0.95 (0.94–0.96)

^a^Age at diagnosis (a quadratic term was added because of its nonlinearity), sex, dependency, metropolitan residency, regions, depression, anxiety, diagnosis of chronic pain, neuropathic pain, and musculoskeletal pain were adjusted in the model.

Abbreviations: CI, confidence interval; OUD, opioid use disorder; PRR, prevalence relative ratio

**Fig 3:**
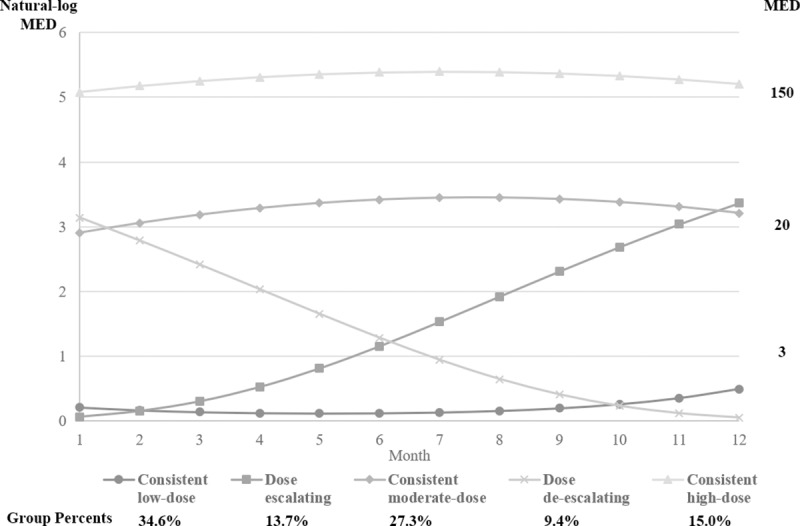
Trajectories of mean daily MED in milligrams prescribed in each month for the 12 months preceding diagnosis of OUD or overdose OD in adults who filled at least 1 opioid prescription. In each subfigure, lines represent types of dose trajectory group, and for each line, each point represents mean daily MED of prescription opioids per month. The scale on the left and right side of the figure was natural logarithm of MED and actual MED, respectively. MED, morphine equivalent dose; OD, overdose; OUD, opioid use disorder.

Among adults with incident OUD or overdose, we identified 5 groups with distinct 12-month prescribed opioid dose trajectories before the outcome (Fig 3). These 5 groups, categorized based on their mean daily MED use of prescription opioids per month, included patients with consistent low-dose use (<3 mg MED), consistent moderate-dose use (20 mg MED), escalating dose use (<3 to 20 mg MED), de-escalating dose (20 to 3 mg or less MED), and consistent high-dose use (150 mg MED). The dose trajectory groups differed significantly for all the demographics, as well as select pain and mental conditions (Table 3).

**Table 3:** Characteristics of defined trajectories of prescribed opioid dose within 12 months before diagnosis of OUD or overdose, 2005–2016.

Characteristic	Percentage (%) of OUD Patients With ≥1 Prescription Opioid Filled Within the Year Before Diagnosis (*n* = 145,609)					
	Low Dose	Escalating Dose	Moderate Dose	De-escalating Dose	High Dose	*P* Value for Group Difference^a^
Total sample size (*n*)	50,464	19,932	39,868	13,697	21,648	
**Age, y**						< .0001
18–30	41.0	17.3	10.4	15.9	6.3	
31–40	18.0	21.4	21.2	19.7	17.6	
41–50	18.8	25.9	29.4	25.9	31.0	
51–64	22.1	35.4	38.9	38.5	45.1	
**Males**	52.3	46.2	43.2	45.7	46.6	< .0001
**Insurance holder status**						< .0001
Dependent	53.5	45.3	46.3	48.2	52.3	
Employee	46.5	54.7	53.7	51.8	47.7	
**Living in metropolitans**	85.1	83.7	82.2	83.3	84.7	< .0001
**Region**						< .0001
Northeast	18.3	13.2	12.8	14.5	16.9	
Central	19.3	18.3	20.5	19.5	19.0	
South	44.5	48.1	44.3	47.3	37.8	
West	17.9	20.3	22.4	18.7	26.3	
**Pain conditions**						
Chronic	39.7	80.7	83.4	77.0	89.6	< .0001
Musculoskeletal	65.1	90.3	90.6	87.4	91.7	< .0001
Neuropathic	16.0	34.4	38.5	35.4	44.5	< .0001
**Mental disorders**						
Depression	32.8	37.3	36.4	37.2	37.0	< .0001
Anxiety	20.9	25.5	22.0	25.1	18.9	< .0001
**Prescription opioid use**						< .0001
Short-acting only	94.4	74.6	69.8	76.6	16.7	
Long-acting only	0.7	1.4	0.9	1.9	3.3	
Short and long-acting	5.0	24.0	29.3	21.5	79.9	
**High dose**						
≥90 mg/day MED in a month	2.2	23.4	28.8	22.6	99.7	< .0001

^a^Determined by chi-squared test.

Abbreviations: MED, morphine equivalent dose; OUD, opioid use disorder

Across the 5 identified opioid dose groups in each cohort, there was variation in the proportion of patients with any monthly mean daily dose above 90 mg MED before OUD or overdose diagnosis (Table 3). The consistent low-dose use group showed the smallest proportion (2.2%) of patients who ever exceeded the high-dose threshold, whereas the consistent high-dose use group had the highest proportion (99.7%). Overall, 72.2% of patients with OUD or overdose who filled opioid prescriptions were never prescribed a mean daily dose of 90 mg MED or more during any month of the 12 months before OUD or overdose diagnosis.

## Discussion

To our knowledge, the present study using US national, commercial insurance claims data is among the first to provide population-based data on prescription opioid use prior to incident OUD or opioid overdose. Our results provide important insight into the role of commonly used high-risk use criteria in identifying patients at risk for OUD or overdose. We found that more than one-third of patients with incident OUD or overdose had not filled any opioid prescriptions in the year before their diagnosis. More than half of those without opioid prescriptions were young males between 18 and 30 years of age. The proportion of patients whose new OUD or overdose diagnosis was not attributable to their own prescription opioids increased by 86% (after adjusting for patient characteristics) during the study period, resulting in 42% of patients with OUD or overdose without opioid prescriptions in 2016. Our findings are largely consistent with growing literature indicating that prescription opioid use is decreasing, which appears to extend to those populations who are at risk for OUD [6,28]. The findings also echo recent data reported by the US Centers for Disease Control and Prevention (CDC) revealing an increase in heroin overdose deaths starting in 2010 and deaths involving synthetic opioids (mainly fentanyl) in 2013 [29]. Taken together, these findings imply that an increasing proportion of patients with OUD or overdose might have used nonprescription opioids to achieve pain control or to enhance euphoric effects before the disease diagnosis.

There are different pathways regarding how prescribed opioids can lead to overdoses from heroin versus overdoses from prescription opioids. Many prescription opioid users transition to heroin injection after having nonmedical use of prescription opioids, such as misusing prescription opioids (e.g., Oxycontin) and using opioid pills not prescribed for them [9]. Transitioning to heroin is ostensible when patients’ tolerance to prescription opioids grows or they have difficulty in accessing prescriptions [30]. On the other hand, the pathways to prescription OUD and overdose appear to largely originate from inadequate pain control, followed by recreational use, nonmedical use of prescription opioids, and use of opioids for the relieving tension and emotional stress, rather than pain [10,31].

Among patients who did fill opioid prescriptions during the year before their OUD or overdose diagnosis, the present study found 5 distinct trajectories of prescribed opioid doses. Some of these dose trajectories may be not necessarily clinically intuitive. For example, 34.6% of patients with OUD or overdose received only a low dose of opioids less than 3 mg/day MED, and 9.4% had de-escalated to the low dose of opioids before the diagnosis. Although some individuals can be susceptible to even small opioid doses (e.g., because of genetic differences in metabolizing enzymes) [32], literature has suggested that the minimum dose for increased risk of opioid overdose is 20 mg/day MED [33]. Thus, the observed dose trajectories reflect pathological pathways incompletely, suggesting a need for a better understand and capture of the drugs and substances that may have contributed to the OUD or overdose. This might include combinations of prescription drugs such as emerging reports of concomitant use of gabapentinoid leading to overdoses events [34–36], increasing manipulation of abuse-deterrent formulations such as observed with Opana [37], and rising supplementation of prescribed supply with prescription or illicit opioids from other sources. This might include heroin use, which has increased 5-fold in recent years [38].

Importantly, our study observed that 72% of patients receiving opioid prescriptions prior to OUD or overdose were prescribed a mean daily opioid dose below the current high-risk threshold of 90 mg MED. Our findings suggest that even among patients who are receiving opioids as part of their treatment plan, only a small proportion at risk for OUD or overdose will be recognized when relying on current risk stratification algorithms. Although the CDC has recommended avoiding increasing the daily dosage of prescribed opioids to 90 mg MED, there is a controversy over what constitutes a “high dose” [39]. Therefore, dose considerations alone provide a limited understanding of opioid risk without considering mental disorders, histories of trauma or substance use, and other risk factors. Our recent research suggested the Centers for Medicare and Medicaid Services (CMS) opioid overutilization criteria, which also include the 90 mg MED/day ceiling for risk identification, seemed not a good clinical marker for identifying Medicare patients at risk for OUD or overdose [15]. Replicating the similar design in a privately insured population will elucidate the accuracy of the high-dose criterion to assign patients into high-risk strata, especially in light of the increasing adoption and/or enforcement of the 90 mg MED ceiling in clinical practice [11,13].

Several strengths of this study are noteworthy. Our findings complement the existing literature by adding to the understanding of prior prescription opioid use before OUD or overdose. Use of administrative claims data yielded a large sample of adults with newly diagnosed OUD or overdose. Using more than a decade of accumulated data representing the US commercially insured population revealed secular trends in prescription opioid use before OUD or overdose diagnosis. Finally, the use of GBTMs allowed the identification of trajectories of prescription opioid doses among patients with OUD or overdose.

Several limitations warrant mention. First, reliance on reimbursed pharmacy dispensing events did not capture prescriptions paid out of pocket. Prescription drug monitoring program data may further inform our findings, even though there is limited evidence that privately insured populations with comprehensive drug coverage commonly resort to cash payment for opioids. Second, our claims data had no information on race/ethnicity, income and education, and other important risk factors for OUD or overdose, which may have further informed our multivariable models [10]. Third, we inferred the onset of OUD or overdose based on the first medical encounter with a diagnosis. Because OUD diagnosis occurs oftentimes delayed, the reported opioid dose trajectories should not be interpreted as causal risk factors for the development of OUD or overdose but rather understood as a historical snapshot of prescription opioid use that is available to clinicians when diagnosing OUD or overdose. Also, there is a limitation to the use of ICD codes for identifying OUD, as some clinicians may under- or overreport misuse/abuse in patients with opioid treatment [3]. To our knowledge, there are no studies to validate the ICD code–based algorithm for OUD. The limitation with the ICD code–based algorithm for OUD, as well as a limited 12-month washout period without OUD or overdose, might have prevented us from accurately identifying incident OUD cases. Thus, a proportion of our selected studied incident OUD population might include patients with prevalent cases of OUD that were known but underdiagnosed during the 12-month baseline or whose diagnosis was recorded prior to the baseline period. If the likelihood of being prescribed opioids differs between incident and prevalent OUD cases, our results of low prescription opioid use may be biased. Finally, our study results are only generalizable to privately insured populations and may not extend to patients covered by public insurance or those who are uninsured.

## Conclusion

In this study of US commercially insured adults with incident OUD or overdose, 35.1% had no opioid prescription fills in the year before diagnosis, and the proportion increased in more recent years. Patients with OUD or overdose exhibited substantial heterogeneity in individual prescription opioid dose trajectories in the year preceding the diagnosis, with the majority receiving prescribed daily doses below the guideline-recommended high-risk threshold of 90 mg MED. Our findings suggest that the vast majority of patients at risk for OUD or overdose will not be identified if solely relying on currently employed prescription-based metrics. Further studies are needed that examine whether the absence of prescription fills was associated with overdoses from illicit opioids and the relationship between dose trajectories with incident OUD or overdose in this new era of decreasing access to prescription opioids.

## Supporting information

S1 STROBE Checklist STROBE, strengthening the reporting of observational studies in epidemiology. (DOC)

S1 Text Prespecified analysis plan. (DOCX)

S1 Table Study prescription opioids approved by the US food and drug administration for use in the US market between 2005 and 2016. (DOCX)

S2 Table Criteria used to decide an optimal solution for number of latent groups among patients with opioid use disorder or overdose who received prescription opioids. (DOCX)

S1 Fig Secular trends in the proportion of adults with OUD or overdose without prescription opioid fills within 12 months before the diagnosis (using ICD-9 codes only), overall and by age groups, from January 1, 2006, to September 30, 2015. OUD, opioid use disorder.
(TIF)

## Abbreviations

CDC: Centers for Disease Control and Prevention
CI: confidence interval
CMS: Centers for Medicare and Medicaid Services
GBTM: group-based trajectory model
MED: morphine equivalent dose
OUD: opioid use disorder
PRR: prevalence relative ratio
STROBE: Strengthening the Reporting of Observational Studies in Epidemiology
