# Supplementary material for: Trends in prescription opioid use and dose trajectories before opioid use disorder or overdose in US adults from 2006 to 2016: A cross-sectional study
Source: PLoS Med. 2019 Nov 5;16(11):e1002941. doi: 10.1371/journal.pmed.1002941 (PMC6830744; doi:10.1371/journal.pmed.1002941)
Supplement: S1 Text — (DOCX) [file pmed.1002941.s002.docx]

**Title: Trajectories and Determinants of High-Risk Prescription Opioid Use**

**Background and Significance**

Opioid use disorder (OUD) has contributed to a substantial number of opioid-related overdose and deaths.^1,2^ Opioid use disorder manifests as a problematic pattern of opioid use that leads to clinically significant impairment or stress.^3^ OUD is defined based on 11 symptoms involving excessive opioid use and its effect on physical on social function within a 12-month period.^3^ An estimate of 2 million individuals in the United States (US) had OUD, with the majority being adults aged 18 to 64 years.^4^ The prevalence of diagnosed OUD in adult populations between 2006 and 2011 tripled (from 0.07% to 0.19%),^5^ in part due to the increased availability of prescription opioids during the same period.^6^

Many studies have explored prescription opioid use patterns and their association with adverse opioid events, including OUD, opioid-related overdoses, and deaths among adults. Although providing valuable information, some of the prior studies have relied on surveys of OUD patients who self-reported prescription opioid use, which is subject to report bias. Other studies used administrative claims data to assess opioid use at a certain moment in time (e.g., baseline), failing to consider that opioid use is likely to change over time and exhibit heterogeneous patterns across individuals.^7-9^ Further, none of the prior studies was conducted during this new era of increasingly restricted access to prescription opioids in the U.S., which may yield prior metrics that focus on specific patient behaviors, such as seeking early refills and doctor shopping, less useful in identifying high-risk opioid users.^10,11^ It remains unclear whether prescription opioid use has been changed following the implementation of the opioid-related policies.

In this study, we propose a longitudinal assessment of prescription opioid utilization towards the diagnosis of OUD or overdose to identify clusters of patients following similar patterns of drug use. Providing a granular picture of opioid use and dose trajectories prior to OUD or overdose manifestation is important for two reasons. First, empirical data on prescription opioid use help clinicians conceptualize the development of prescription opioid use across high-risk opioid users before the disease onset. Because of a limited time window (e.g., 12 months) of patient history data available for routine clinical assessment, our study that summarizes prescription opioid trajectories during the 1-year window before OUD or overdose diagnosis may assist clinicians in timely identification of typical or atypical high-risk opioid use patterns that require further management and intervention. Second, our study using multiple years (2006-2016) of prescription dispensing data will allow for understanding whether prescription opioid use patterns have been changed over time, during which many policies and programs aimed at limiting opioid prescribing were implemented.

**Project Objectives:** Our primary objectives are twofold. First, the study aims to provide a comprehensive description of how prescription opioids have been used toward the diagnosis of serious opioid-related adverse events (i.e., OUD or overdose). Second, we will test whether the opioid usage has been changed over time in light of increasing restriction on access to prescription opioids in the US. Third, we will examine risk factors associated with high-risk opioid use. We will use 11 years (2005-2016) of administrative claims data of privately-insured beneficiaries linked to their prescription drug events to accomplish the following aims:

**Aim 1**: Examine the prevalence and trend of prescription opioid use in the 12 months prior to the diagnosis of OUD or overdose among adults

**Aim 2**: Examine trajectories of prescription opioid use and dose 12 months prior to the diagnosis of OUD or overdose among adults who filled opioid prescriptions prior to diagnosis.

**Proposed Methods**

**Research design:** This study proposes a cross-sectional study design among adults aged between 18 and 64 years old who were newly diagnosed with OUD or opioid-related overdose. We will examine the prevalence and trajectories of prescription opioid use over 12 months preceding an incident OUD or overdose. This period is chosen to emulate the real-world clinical practice where a limited time window of patient history data is often available for routine clinical assessment.

**Data source:** We used the 2005-2016 Truven MarketScan databases of Commercial and Medicare supplement claims to conduct a cross-sectional study of individuals across the age spectrum to assess 11-year trends of OUD/OD incidence and prescription opioids and adjuvant analgesics among the patients with OUD/OD. The Truven database consists of more than 350 self-insured employers and health plans, including approximately 46.9 million youths and 135.9 million adults in the commercial claims datasets, and 21.5 million older adults participating in Medicare supplemental insurance programs in the US between 2005 and 2016. The Truven database has been widely used to evaluate trends in incidence or prevalence of diseases (including OUD and OD) and prescription drug use among different age groups. The files contain enrollee billing records for inpatient and outpatient encounters and pharmacy filled prescriptions, as well as enrollee demographics and enrollment status. The inpatient and outpatient records include details on procedures and diagnoses, coded using the International Classification of Disease, Ninth or Tenth Revision, Clinical Modification (ICD-9-CM or ICD-10-CM).

**Sample selection:** Eligibility criteria: 1) patients who were aged 18-64 years at the time of a new OUD or overdose diagnosis, defined as having no history of either diagnosis during the 12 months preceding the date of the first recorded OUD or overdose (ie, index date); 2) patients who had continuous health plan enrollment for 12 months before the index date; and 3). Exclusion criteria: 1) ≥1 outpatient or inpatient encounters with OUD or overdose diagnosis during the 12-month pre-index period; 2) receiving cancer and hospice care during the 12-month pre-index period.

OUD was measured as having at least 1 inpatient or outpatient encounter claim with an ICD-9-CM code of 304.0x, 304.7x, 305.5x, or an ICD-10-CM code of F11.xx and patients with opioid overdose with ICD-9-CM code 965.0x, or E850.0 to E850.2 or an ICD-10-CM code of T40.0xx to T40.4xx (where the 'xx' placeholders contained X1 or X4) or T40.601, T40.604, T40.691, T40.694 in any diagnostic position.^12^ When identifying patients with incident OUD or overdose, we excluded ICD-10-CM codes that indicated “in remission” (e.g., F11.x1) or “subsequent encounter” (e.g., F40, 0X1D) of OUD or overdose.

**Prescription opioids**: Study prescription opioids include those approved by the US food and drug administration for use in the US market between 2005 and 2016. We capture the studied prescription opioids through the MarketScan pharmacy files included agents. We excluded injectable opioids primarily used in inpatient settings where dispensing information is unavailable due to capitation-based reimbursement, rectal dosage forms, which are rarely used, and buprenorphine because it is mostly used for OUD treatment. Tapentadol and opioid prescriptions with missing strength (both < 1% of the opioid prescription claims) were included in the analysis of opioid prescription receipt before OUD or overdose but excluded in dose trajectory analysis due to the lack of a conversion factor for estimating morphine milligram equivalent (MME) dosage.^13^ We will convert the dose of each prescribed opioid fill to MME using a standard formula used by the Center for Medicare and Medicaid Services.^13^

**Risk factors**: We measured variables that had previously reported associations with OUD or overdose. ^14-17^ The variables are listed in Table 1.

| **Table 1: Summary of measures for risk factors** | | |
| --- | --- | --- |
| **Factors** | **Variables** | **Data Sources** |
| Demographics | age, biological sex, living in a metropolitan area (yes vs no), region (Northeast, Central, South or West US), Insurance holder status (employee vs. dependent) | Truven annual summary enrollment file |
| Pain conditions^a^ | - *Chronic pain*: requiring ≥1 inpatient or outpatient diagnoses in “highly likely” or ≥2 inpatient or outpatient diagnoses in “likely” that are separated by ≥30 days(ICD-9-CM: High likely: 338.2,338.4; Likely: 307.80, 338.0, 719.41, 719.45, 719.46, 719.47, 719.49, 720.0, 720.2, 720.9, 721.0, 721.1, 721.2, 721.3, 721.4, 721.6, 721.8, 721.9, 722, 723.0, 723.1, 723.2, 723.3, 723.4, 723.5, 723.7, 723.8, 723.9, 724, 729.0, 729.1, 729.2, 729.4, 729.5)   *Musculoskeletal pain:* requiring ≥1 inpatient or ≥ 1 outpatient diagnoses (ICD-9-CM: 715, 716,719, 723, 724 [except 724.3,724.4], 727,728,729 [except 729.2]).   - *Neuropathic pain*: requiring ≥1 inpatient or ≥ 1 outpatient diagnoses (ICD-9-CM: 053.12,053.13,337.2, 338.0, 350.1,350.2,355.4,355.71,355.9, 356, 724.3,724.4, 729.2) | Truven inpatient services and outpatient services files |
| Mental health conditions^a^ | - *Depression:* requiring ≥1 inpatient or ≥ 1 outpatient diagnoses (ICD-9-CM: 296.2,296.3, 296.5, 296.6, 296.89,298.0, 300.4, 309.1, 311) - *Anxiety:* requiring ≥1 inpatient or ≥ 1 outpatient diagnoses (ICD-9-CM: 293.84,300.0, 300.10, 300.2, 300.3, 300.5, 300.89, 300.9,308,309.81, 313.0-313.3, 313.82, 313.83) | Truven inpatient services and outpatient services files |
| Prescription opioid use | - *Chronic opioid use*, defined as 70 days or more in a 90-day period - *High dose of opioid use:* assessed in terms of a cautionary dose of 50mg/day MED or grater and a high-risk dose of 90mg/day MED or greater in any 30-day period and - *Type of opioid use*, grouped as short-acting only, long-acting only, and both short- and long-acting dosage forms. | Truven outpatient drug claims files and RED BOOK ® |

^a^ All ICD-9-CM codes are converted to the corresponding ICD-10-CM codes.

**Data analyses:** Our overall approach to the analysis will focus on descriptive analyses, group-based trajectory modeling, and multivariable models. All tests are at two-sided with statistical significance at P < .05. The following details the analyses planned for each aim.

***Aim 1: Examine the prevalence and trend of prescription opioid use in the 12 months prior to the diagnosis of OUD or overdose among adults***

*Descriptive analysis*: we will calculate summary statistics (including mean, median, and range) of prescription opioid use in the 12 months prior to the diagnosis of incident OUD or overdose, in the entire sample and stratified by demographics, pain conditions, mental health conditions and prescription opioid use. We reported the annual crude prevalence of prescription opioid fills in the 12 months prior to the OUD or overdose diagnosis from 2006 to 2016 in the overall eligible patients and in 4 age groups: 18-30, 31-40, 41-50, and 51-64 years according to the age at the beginning of the corresponding year. To describe trends in the receipt of prescription pain medications among patients with incident OUD/OD, we calculated a percentage change for each outcome of interest from 2006 to 2016. After the ICD-10 coding system, the number of opioid-related diagnosis codes increased from 20 ICD-9-CM codes to 100 ICD-10-CM, resulting in a dramatic increase in the number of individuals diagnosed with OUD or overdose. Thus, we conducted a sensitivity analysis using ICD-9-CM codes only to test secular trends between 1/1/2006 to 9/30/2015 to test whether the trends differ from the period of 1/1/2006-12/31/2016, during which ICD-10 coding system has been implemented.

*Trend analysis*: To test the temporal trend, we fitted a modified Poisson regression with use of prescription opioid (yes or no) as the dependent variable and each calendar year (dummy variable) as the main independent variable, adjusting for other covariates listed in Table 1. The coefficients of these yearly dummy variables represent changes in the proportion of OUD patients without opioid prescription fill for a given year compared with the reference year of 2006. We reported prevalence relative ratios (PRRs) and their 95% confidence intervals (CI) of each independent variable.

***Aim 2: Examine trajectories of prescription opioid dose 12 months prior to the diagnosis of OUD or overdose among adults who filled opioid prescriptions prior to diagnosis.***

In the sample of adults with OUD or overdose filling opioid prescriptions in the 12 months prior to the diagnosis, we used a Group-based trajectory modeling (GBTM), a latent class analysis, to describe the natural history of prescription opioid dose before the disease onset.^18^ The change of prescription opioid dose over time is its developmental trajectory. According to the assumption of GBTM, the study population (i.e., patients with OUD or overdose) has a finite number of clusters of individuals, and each cluster follows approximately a similar trajectory of opioid dose before the disease diagnosis. GBTM provides an empirical means of identifying clusters of individuals following typical and atypical development, which can help clinician conceptualize the change of clinical conditions or treatments.^18^

**GBTM estimation**s are generated by maximum likelihood estimation. Equation 1 describes the likelihood of an individual’s observed repeated prescription opioid dose is composed of two elements –$\pi_{j}$, which denotes the probability of trajectory group membership*; and* $P^{j}\left( Y_{i} \right)$, which denotes the probability of the observed outcome data given group membership

$P\left( Y_{i} \right)$=$\sum_{j} \pi_{j}P^{j}(Y_{i})$ ………………………………. Equation 1^18^

$Y_{i}$= prescription opioid dose trajectory data for an individual *i* over the 12 months prior to OUD or overdose diagnosis

$P^{j}\left( Y_{i} \right)$= probability of $Y_{i}$ if belonging to group *j*

$\pi_{j}$=probability of trajectory group membership *j=1,…, J*

The group membership probabilities are estimated by a multinomial logit function. The conditional probability of $Y_{i}$ given group membership j is indexed by the unknown parameter vector j, which also determines the shape of the group-specific trajectory. The conditional probability of $Y_{i}$ (i.e., outcome measure) is determined with a polynomial function of time. Depending on the distribution of the outcome measure, GBTM can model the data in various forms, including normal, censored normal, binary, or Poisson distribution. Because our prescription opioid dose data cluster within a certain range (0-170 MMEs), with some outlier points, the probability of outcome trajectory data is assumed to follow the censored normal distribution.

**Evidence of clusters in GBTM**: We will use GBTM to map the longitudinal pattern of prescription opioid use and to identify clusters of initiators with similar trajectories using Proc Traj in SAS version 9.4. (download from [www.andrew.cmu.edu/~bjones](http://www.andrew.cmu.edu/~bjones)) ^18^ Following recommended procedures, we will test the model with three different polynomial forms, including linear, quadratic, and cubic terms to determine the best trajectory shape that fit the prescription opioid dose data. We will then determine the optimal number of trajectory groups based on (1) Bayesian information criteria (BIC) and Akaike information criterion (AIC), with a lower BIC/AIC indicating a better model fit; (2) model adequacy, evidenced by an average posterior probability of at least 0.7 in each group identified; (3) sufficient group size constituting at least 5% of the total sample; and (4) clinical relevance. After identifying trajectory groups for each opioid use metric and the composite measure, we will use chi-square tests to explore whether baseline patient characteristics and pain diagnoses differ among groups.

**Reference**

1. Rudd RA, Seth P, David F, Scholl L. Increases in Drug and Opioid-Involved Overdose Deaths - United States, 2010-2015. *MMWR Morbidity and mortality weekly report.* 2016;65(5051):1445-1452.

2. Rudd RA, Aleshire N, Zibbell JE, Gladden RM. Increases in drug and opioid overdose deaths--united states, 2000-2014. *MMWR Morbidity and mortality weekly report.* 2016;64(50-51):1378-1382.

3. Hasin DS, O'Brien CP, Auriacombe M, et al. DSM-5 criteria for substance use disorders: recommendations and rationale. *Am J Psychiatry.* 2013;170(8):834-851.

4. Florence CS, Zhou C, Luo F, Xu L. The Economic Burden of Prescription Opioid Overdose, Abuse, and Dependence in the United States, 2013. *Med Care.* 2016;54(10):901-906.

5. Kirson NY, Shei A, Rice JB, et al. The Burden of Undiagnosed Opioid Abuse Among Commercially Insured Individuals. *Pain medicine.* 2015;16(7):1325-1332.

6. Guy GP, Jr., Zhang K, Bohm MK, et al. Vital Signs: Changes in Opioid Prescribing in the United States, 2006-2015. *MMWR Morbidity and mortality weekly report.* 2017;66(26):697-704.

7. Han B, Compton WM, Jones CM, Cai R. Nonmedical Prescription Opioid Use and Use Disorders Among Adults Aged 18 Through 64 Years in the United States, 2003-2013. *Jama.* 2015;314(14):1468-1478.

8. Han B, Compton WM, Blanco C, Crane E, Lee J, Jones CM. Prescription Opioid Use, Misuse, and Use Disorders in U.S. Adults: 2015 National Survey on Drug Use and Health. *Annals of internal medicine.* 2017;167(5):293-301.

9. West NA, Severtson SG, Green JL, Dart RC. Trends in abuse and misuse of prescription opioids among older adults. *Drug and alcohol dependence.* 2015;149:117-121.

10. Chang HY, Murimi I, Faul M, Rutkow L, Alexander GC. Impact of Florida's prescription drug monitoring program and pill mill law on high-risk patients: A comparative interrupted time series analysis. *Pharmacoepidemiol Drug Saf.* 2018.

11. Moyo P, Simoni-Wastila L, Griffin BA, et al. Impact of prescription drug monitoring programs (PDMPs) on opioid utilization among Medicare beneficiaries in 10 US States. *Addiction.* 2017;112(10):1784-1796.

12. Green CA, Perrin NA, Janoff SL, Campbell CI, Chilcoat HD, Coplan PM. Assessing the accuracy of opioid overdose and poisoning codes in diagnostic information from electronic health records, claims data, and death records. *Pharmacoepidemiology and drug safety.* 2017;26(5):509-517.

13. The Centers for Medicare and Medicaid Services. Opioid Oral Morphine Milligram Equivalent (MME) Conversion Factors. In:2018.

14. Edlund MJ, Steffick D, Hudson T, Harris KM, Sullivan M. Risk factors for clinically recognized opioid abuse and dependence among veterans using opioids for chronic non-cancer pain. *Pain.* 2007;129(3):355-362.

15. Edlund MJ, Martin BC, Fan MY, Devries A, Braden JB, Sullivan MD. Risks for opioid abuse and dependence among recipients of chronic opioid therapy: results from the TROUP study. *Drug and alcohol dependence.* 2010;112(1-2):90-98.

16. Wilsey BL, Fishman SM, Tsodikov A, Ogden C, Symreng I, Ernst A. Psychological comorbidities predicting prescription opioid abuse among patients in chronic pain presenting to the emergency department. *Pain medicine.* 2008;9(8):1107-1117.

17. Cochran BN, Flentje A, Heck NC, et al. Factors predicting development of opioid use disorders among individuals who receive an initial opioid prescription: mathematical modeling using a database of commercially-insured individuals. *Drug and alcohol dependence.* 2014;138:202-208.

18. Nagin DS, Odgers CL. Group-based trajectory modeling in clinical research. *Annual review of clinical psychology.* 2010;6:109-138.
