# Supplementary material for: Trends in prescription opioid use and dose trajectories before opioid use disorder or overdose in US adults from 2006 to 2016: A cross-sectional study
Source: PLoS Med. 2019 Nov 5;16(11):e1002941. doi: 10.1371/journal.pmed.1002941 (PMC6830744; doi:10.1371/journal.pmed.1002941)
Supplement: S1 Table — (DOCX) [file pmed.1002941.s003.docx]

S1 Table. Study prescription opioids approved by the US food and drug administration for use in the US market between 2005 and 2016

| **Generic Drug Name** | **Controlled Substance Schedule** |
| --- | --- |
| Butorphanol | IV |
| Codeine | II |
| Dihydrocodeine | II |
| Fentanyl | II |
| Hydrocodone | II |
| Hydromorphone | II |
| Levorphanol | II |
| Meperidine | II |
| Methadone | II |
| Morphine | II |
| Nalbuphine | Not controlled |
| Opium | II |
| Oxycodone | II |
| Oxymorphone | II |
| Pentazocine | IV |
| Propoxyphene^a^ | IV |
| Remifentanil | II |
| Sufentanil | II |
| Tramadol | IV |

^a^Propoxyphene was withdrawn from the US market in 2010.
