# Supplementary material for: Trends in prescription opioid use and dose trajectories before opioid use disorder or overdose in US adults from 2006 to 2016: A cross-sectional study
Source: PLoS Med. 2019 Nov 5;16(11):e1002941. doi: 10.1371/journal.pmed.1002941 (PMC6830744; doi:10.1371/journal.pmed.1002941)
Supplement: S2 Table — (DOCX) [file pmed.1002941.s004.docx]

S2 Table. Criteria used to decide an optimal solution for number of latent groups among patients with opioid use disorder or overdose who received prescription opioids^a^

|  | **Among Patients with Opioid Use Disorder Who Received Prescription Opioids** | | | |
| --- | --- | --- | --- | --- |
| **Number of groups** | **BIC** | **AIC** | **Posterior probability** | **Group size (% of total sample)** |
| 1 | -3,282,824 | -3,282,804 | Group 1: 1.000 | 100 |
| 2 | -2,724,417 | -2,724,377 | Group 1: 0.995  Group 2: 0.995 | 51.6  48.4 |
| 3 | -2,622,322 | -2,622,262 | Group 1: 0.992  Group 2: 0.967  Group 3: 0.979 | 42.7  27.3  30.0 |
| 4 | -2,547,575 | -2,547,496 | Group 1: 0.990  Group 2: 0.968  Group 3: 0.969  Group 4: 0.975 | 39.1  12.7  26.8  21.5 |
| **5** | **-2,477,970** | **-2,477,871** | **Group 1: 0.987**  **Group 2: 0.970**  **Group 3: 0.973**  **Group 4: 0.971**  **Group 5: 0.974** | **34.6**  **13.7**  **27.3**  **9.4**  **15.0** |
| 6^b^ | -2,450,945 | -2,450,826 | Group 1: 0.986Group 2: 0.971  Group 3: 0.963  Group 4: 0.972  Group 5: 0.972  Group 6: 0.974 | 33.7  12.5  8.926.1  4.9  13.9 |
| 7 | -2,417,117 | -2,416,979 | Group 1: 0.978  Group 2: 0.959  Group 3: 0.967  Group 4: 0.949  Group 5: 0.972  Group 6: 0.978  Group 7: 0.978 | 29.8  9.6  6.4  10.5  25.0  6.0  12.7 |

Abbreviations: AIC, Akaike Information Criteria; BIC, Bayesian Information Criteria.

^a^ Following recommended procedures, we fitted group-based trajectory models with 1 to 7 groups, and each model was tested with liner, quadratic, and cubic terms to determine the best shapes that fit the data. We then determined the optimal number of trajectory groups based on (1) BIC and AIC, with a lower BIC/AIC indicating a better model fit; (2) model adequacy, evidenced by an average posterior probability of at least 0.7 in each group identified; (3) sufficient group size that constituted at least 5% of the total sample; and (4) clinical relevance. These procedures indicated that a model with 5 trajectories was optimal.

^b^ Both models (ie, model with 5 trajectories and model with 6 trajectories) have a good model fit with relative low BIC and AIC, model adequacy (posterior probability of at least 0.7 in each group identified) and sufficient group size (all groups identified constituted at least 5% of the total sample). Clinical relevance: the model with 5 trajectories represents a valid clinical picture by identifying 5 distinct groups. The model with 6 trajectories is suboptimal because both groups 5 and 6 identified in this model represent a similar group of individuals with high dose (≥90-mg morphine equivalent dose) prescription opioid use.
