# Supplementary figures and images for: Trends in prescription opioid use and dose trajectories before opioid use disorder or overdose in US adults from 2006 to 2016: A cross-sectional study
Source: PLoS Med. 2019 Nov 5;16(11):e1002941. doi: 10.1371/journal.pmed.1002941 (PMC6830744; doi:10.1371/journal.pmed.1002941)

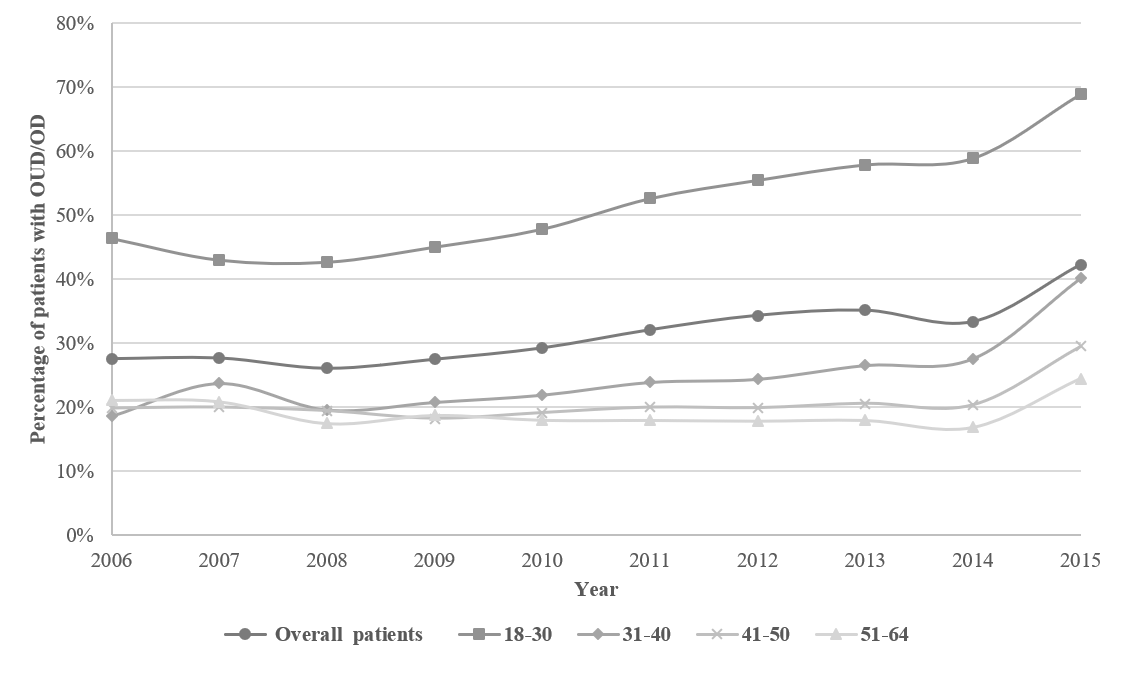

Supplement: S1 Fig — OUD, opioid use disorder. (TIF) [file pmed.1002941.s005.tif]
